# Study of the correlation between central venous oxygen saturation and venous saturation from the antecubital vein in severe sepsis/septic shock patients

**DOI:** 10.1186/cc10862

**Published:** 2012-03-20

**Authors:** K Piyavechviratana, W Tangpradubkiet

**Affiliations:** 1 Phramongkutklao Hospital, Bangkok, Thailand

## Introduction

Early goal-directed therapy has been used for severe sepsis and septic shock in the ICU to achieve a balance between systemic oxygen delivery and oxygen demand before global tissue hypoxia develops and proceeds to multiorgan failure. One of the resuscitation end points includes normalized values for central venous oxygen saturation (ScvO_2_) that needs insertion of a central venous catheterization, which is still impractical in small-to-medium-sized hospitals in Thailand. The purpose of this study was to examine whether the venous oxygen saturation from the antecubital vein has correlation with the central venous oxygen saturation or can be applied instead of the central venous oxygen saturation.

## Methods

This was an observational study performed during 4 July 2007 to 31 March 2009 in the 10-bed ICU of Pramongkutklao Hospital in severe sepsis or septic shock patients who already had a central venous catheter inserted. Two blood samples were collected and sent to the laboratories for blood gas analysis. We then calculated for the correlation using correlation and linear regression analysis.

## Results

**Figure 1:**
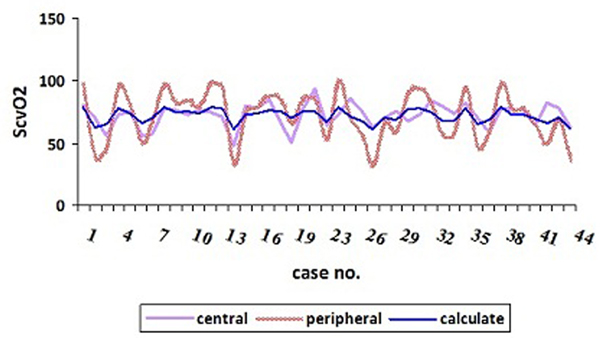
**Central and peripheral oxygen saturation range**.

Of the 44 enrolled patients, 24 were males (54.54%). Mean age was around 69.86 ± 16.819 years. A total of 84.1% was in septic shock. The most common source of infection was pneumonia (38.6%). The central ScvO_2 _and peripheral venous oxygen saturation ranges and means were 46.0 to 93.2%, 31.5 to 99.0% and 71.66 ± 10.39%, 71.18 ± 19.79% respectively. The correlation between ScvO_2 _and antecubital venous oxygen saturation significant *P *value was 0.000: calculated ScvO_2 _= 52.386 + 0.271(peripheral), *R*^2 ^= 0.266. The specificity, sensitivity, positive predictive value and negative predictive value of the predicting equation were 69.23%, 77.41%, 85.71% and 56.25% respectively. The accuracy was 75%. See Figure 1.

## Conclusion

Venous oxygen saturation from the antecubital vein was not the exact value of central venous oxygen saturation but there were significant correlations.

